# Supplementary material for: Chromothripsis during telomere crisis is independent of NHEJ, and consistent with a replicative origin
Source: Genome Res. 2019 May;29(5):737–49. doi: 10.1101/gr.240705.118 (PMC6499312; doi:10.1101/gr.240705.118)
Supplement: Supplemental Material [file supp_gr.240705.118_Supplemental_file_1.zip › contigs/annotated_contigs/DB95/contig.3.DB95_length_229_mean_cov_5.78602620087.docx]

**DB95_length_229_mean_cov_5.78602620087**

```
TTTCTGGG|TGTGTAGTAGTCTATGATATATATATATATATATATATA|TATAT|ATATATATATATATATATATATATATATATATAT
         >chr12:87880579-87880623 - E=3e-06 p=0e+00 >chr14:46954933-46955080 + E=1e-66
ACACTTTTTTTAAATCTAGTCTACCATTGATGGGTATTTAGGTTGATTCCATGTCTTTGCTGTTGTGAATAGTACTACAACGGACATAT

GCATGCATGTGTC|TTTAT|AGTAGAATGATTTATAATCATTCTACTATATACCCA              >chr4:33310214-33310255 - E=2e-07
```
